# The Role of Natural Killer Cells as a Platform for Immunotherapy in Pediatric Cancers

**DOI:** 10.1007/s11912-019-0837-8

**Published:** 2019-09-10

**Authors:** Miriam Santiago Kimpo, Bernice Oh, Shawn Lee

**Affiliations:** Division of Paediatric Oncology, Department of Paediatrics, National University Hospital Singapore, 1E Kent Ridge Road, NUHS Tower Block Level 12, Singapore, 119228 Singapore

**Keywords:** Natural killer cells, Adoptive cell therapy, Tumor microenvironment, CAR-NK

## Abstract

**Purpose of Review:**

We aim to review the most recent findings in the use of NK cells in childhood cancers.

**Recent Findings:**

Natural killer cells are cytotoxic to tumor cells. In pediatric leukemias, adoptive transfer of NK cells can bridge children not in remission to transplant. Interleukins (IL2, IL15) can enhance NK cell function. NK cell-CAR therapy has advantages of shorter life span that lessens chronic toxicities, lower risk of graft versus host disease when using allogeneic cells, ability of NK cells to recognize tumor cells that have downregulated MHC to escape T cells, and possibly less likelihood of cytokine storm. Cytotoxicity to solid tumors (rhabdomyosarcoma, Ewing’s sarcoma, neuroblastoma) is seen with graft versus tumor effect in transplant and in combination with antibodies. Challenges lie in the microenvironment which is suppressive for NK cells.

**Summary:**

NK cell immunotherapy in childhood cancers is promising and recent works aim to overcome challenges.

## Introduction

Survival rates for cancer in children have risen over the years due to collaborative efforts among working groups, advances in treatment technology, and most importantly, improvement in supportive care. However, outcome in many children, especially in the high-risk groups, remains dismal despite multidisciplinary treatment modalities and maximized chemotherapy regimens [1]. Thus, newer treatment options continue to be explored.

Immunotherapy has advanced significantly in the recent years and is beneficial when incorporated into treatment regimens for cancers in both adults and children. Natural killer cells have gained recognition as knowledge regarding their role in cancer surveillance increases. Their therapeutic roles lie in the settings of adoptive cell therapy from allogeneic donors and haploidentical stem cell transplantation [2]. This review aims to give a concise update on the recent developments regarding NK cell therapy in pediatric oncology.

## Natural Killer Cell Biology

**Fig. 1:**
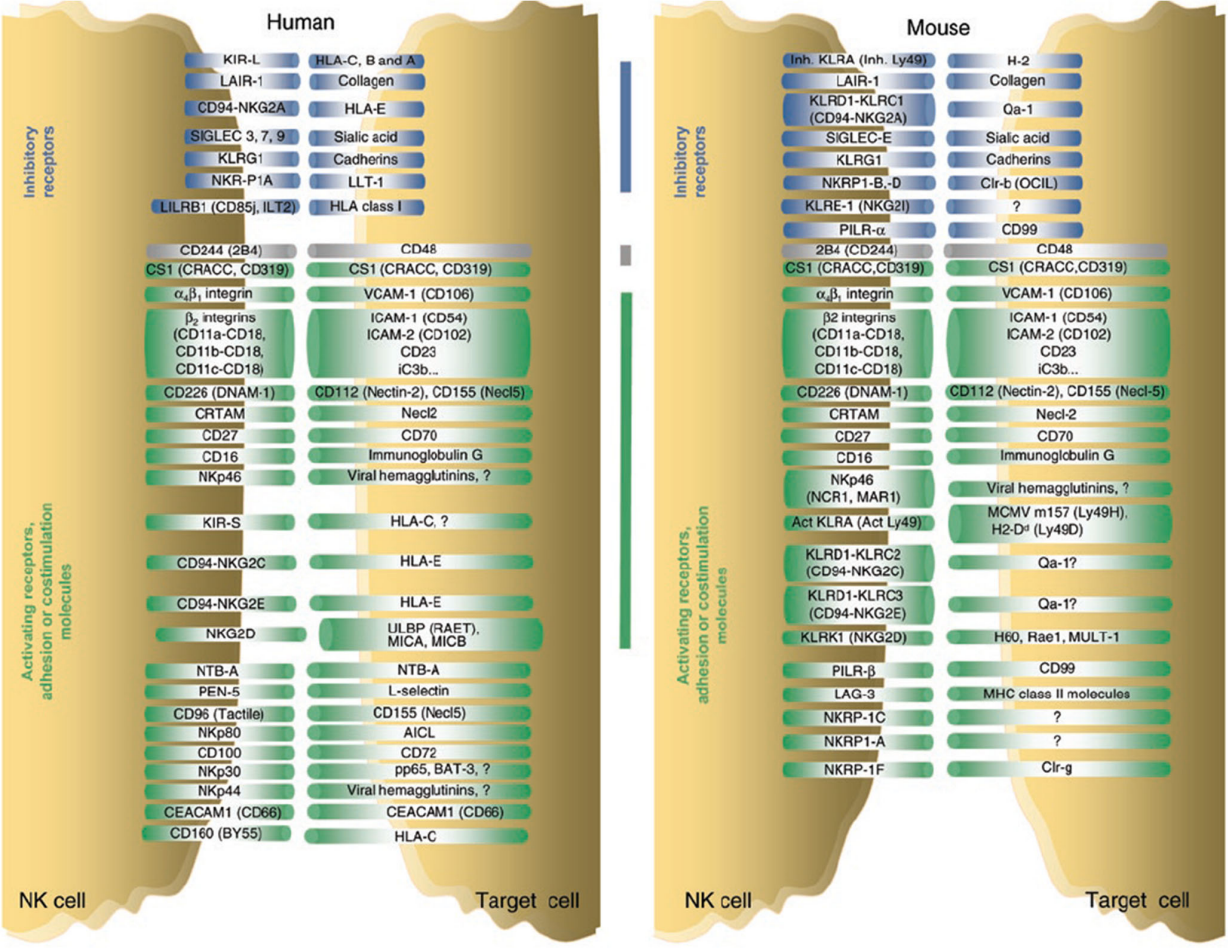
NK cell activation programs result from the integration of multiple activating and inhibitory signals that vary depending on the nature of the interacting cells. These signals involve ITAM (immunoreceptor tyrosine-based activation motif)-bearing molecules and other stimulatory receptors and adhesion molecules, as well as ITIM-bearing inhibitory receptors. Some human (left) and mouse (right) receptor-ligand interactions are depicted here, to illustrate the combinatorial nature of the NK cell interaction repertoire. Cytokines, chemokines, and their receptors are not shown, but are also crucial for the regulation of NK cell functions. Inhibitory receptors are in blue; 2B4, which can act as an activating or an inhibitory molecule, is in gray; other receptors are in green. Vertical lines indicate the receptor-ligand pairs conserved between mice and humans, which consist either of real orthologs (for example, human and mouse NKp46) or examples of convergent evolution (for example, KIR and Ly49). KIR, killer immunoglobulin-like receptors; LIR, immunoglobulin-like transcript; LAIR, leukocyte-associated immunoglobulin-like receptor; SIGLEC, sialic acid binding immunoglobulin-like lectins; KLRG-1, killer cell lectin-like receptor G1; NKR-P1, NK cell receptor protein 1; HLA, human leukocyte antigen; LLT, lectin-like transcript; CRTAM, class I restricted T cell-associated molecule; Necl-2, nectin-like 2; Tactile (also known as CD96), T cell-activated increased late expression; CEACAM1, carcinoembryonic antigen-related cell adhesion molecule 1; PILR, paired immunoglobulin-like type 2 receptor; NTB-A, NK-T-B antigen; CRACC, CD2-like receptor-activating cytotoxic cell; VCAM-1, vascular cell adhesion. Reproduced from Vivier E, et al. “Functions of natural killer cells.” *Nat Immunol*. 2008 May;9 [3]:503-10, by permission from Springer Nature, ©2008

Natural killer cells are effector lymphocytes of the innate lymphoid system that recognizes cells transformed by viruses or cancer and causes their lysis without the need for prior sensitization. Their cytotoxic functions are based on a balance between signals from inhibitory receptors, presence of activating receptors on NK cells, and their ligands on target cells [Fig. 1]. They participate in ADCC through CD16 (FcγRIIIA) which binds to antibodies. Genetic polymorphisms in these receptors are influential in determining response with antibodies such as Rituximab. NK cells can release perforin and granzymes that are directly toxic to cells. They induce apoptosis via Fas and TRAIL pathways. They release cytokines that drive other cells in the immune system such as dendritic cells that are recruited into the tumor bed [4•].

NK cells express CD56 and are negative for CD3. They comprise a diverse population with two more well-defined subsets. CD56bright + CD16 cells are seen mainly in the tonsils and lymph nodes. They secrete cytokines such as gamma interferon but lack perforin which limits their cytotoxic ability. Cells that are CD56dim+ have immunoglobulin receptor CD16 (FCGR3A) and express perforin and KIR (killer inhibitory receptors). They circulate in the peripheral blood and have cytotoxic capabilities. A complex system of activating and inhibitory receptors that recognize ligands in circulating cells helps maintain balance between tolerance and cytotoxicity. Inhibitory receptors recognize native antigens and prevent NK activation. Activating receptors recognize cells that are missing MHC antigens (“missing self”). In the “induced self” hypothesis, cell expression of ligands for activating receptors is induced in the presence of stress. Activating receptors include natural cytotoxicity receptors NKp46, NKp44, and NKp30, DNAM-1 and NKG2D. NKG2D recognizes stress-induced ligands MICA, MICA B, and ULBP1–6. Although part of the innate immune system, NK cells have been identified as having memory functions which is an adaptive response [2, 5•]. Exposure to viruses or cytokines can create memory cells that can unleash increased activity upon re-exposure.

## NK Cell Surveillance in Leukemia

Several observations on NK cells and their role in leukemia predisposition have been made in studies in looking at polymorphisms in KIR genes and their ligands [3]. NK cells are known to kill transformed cells in the body, of which leukemic clones are. A higher number of activating KIR genes which stimulates NK cell cytotoxicity in normal control versus that of children with B ALL suggests the role of KIR genotype in preventing leukemia [6]. Another study also involving children with B-ALL found a correlation between homozygosity for HLA C2 alleles and a higher incidence of late relapse [3]. The strong binding and affinity between C2 and inhibitory KIR2DL1 are postulated to inhibit NK cells and their surveillance for leukemia. Similar associations have also been reported for acute myeloid leukemia (AML), chronic myeloid leukemia (CML), and chronic lymphocytic leukemia (CLL), where a significantly higher incidence of inhibitory KIR phenotypes was found in leukemic patients compared to controls [7]. All these point towards an active and significant role of NK cell surveillance in leukemogenesis.

### Graft Versus Leukemia in Transplants

Further evidence of the role of NK cells in leukemia was noted in studies in allogeneic stem cell transplantation (SCT), especially in acute myeloid leukemia (AML). Significant improvement in AML outcomes is provided by allogeneic stem cell transplantation (SCT). The benefit derived from the graft versus leukemia effect of T cells is complicated by T cells attacking the normal tissues ( graft versus host disease) necessitating its depletion. NK cells are capable of eliminating leukemic cells, restore immunity against viruses early in the posttransplant period, with minimal graft versus host disease [3].

In studies of patients with leukemia who received autologous hematopoietic stem cell transplantation (HSCT), numbers of NK cells correlated with a positive outcome, indicating the anti-tumor potential of NK cells. One study has identified KIR–ligand incompatibility as the major (and possibly only) factor that can be used to predict the outcome of patients with AML who have received HLA-mismatched HSCT [8]. This same study also showed that alloreactive NK cells possibly prevented graft-versus-host disease (GVHD) by eliminating host antigen presenting cells [9]. However, this does not apply to all hematological cancers. While they are protective in AML, KIR–ligand mismatches have not been found to influence transplantation outcome in all leukemias [9]. Further understanding on the mechanism of NK cell activation shows that presence of both iKIR/KIRL mismatch with the recipient as well as multiple activating KIRs can improve outcome by increasing NK alloreactivity in children with B precursor leukemia post T cell-depleted haploidentical transplantation [2].

Hence, it is conceptually attractive to project an extrapolated benefit of NK alloreactivity into the non-transplant setting, with the possibility of further leukemic control by cell-mediated mechanisms in the absence of transplant-related toxicity.

### NK Therapy in Hematologic Cancers

NK cells are typically more effective against leukemia cells compared to solid tumors for a variety of reasons [2, 4•, 10]. Even within leukemias, conventional knowledge describes significant ability of NK cells to eliminate adult AML but less so in ALL. Less knowledge exists as to the significance of NK cell alloreactivity in the context of pediatric leukemia [2]. In line with the data obtained in adult patients, pediatric AML also appears to be a target of alloreactive NK cells [2, 11, 12]. In contrast to adult ALL, pediatric ALL seems to be also susceptible to NK cell-mediated target cell lysis making NK therapy an option for pediatric ALL as well [13, 14].

Miller et al. were the first to report the successful transfer and expansion of haploidentical NK cells in the non-HSCT setting, with five out of 19 patients achieving complete remission, demonstrating potent anti-leukemic effect [15]. Further proof of feasibility of this concept was subsequently demonstrated clearly in the NKAML pilot study at St Jude involving ten AML pediatric patients, where patients had a 2-year event-free survival of 100%. In this study, investigators utilized NK cells purified by CliniMACS with removal of CD3+ cells and enrichment of CD56+ cells. Conditioning regimen was well tolerated, demonstrating safety of this approach as well as efficacy as compared to historical controls [16].

IL-2 is frequently used in combination with adoptive NK cell transfer. This is because early after transplant, NK cells are not optimally functional without the supportive application of IL-2 [17]. Activated NK cells may exert greater immunotherapeutic effects compared to unstimulated cells. A phase I/II comparison study between IL-2-activated NK-DLI and unstimulated NK-DLI in children with leukemia indicated enhanced effector trafficking potential for activated NK cells [18]. Another study was carried out where 16 high-risk refractory adult patients with MDS/AML were given adoptive immunotherapy with IL-2-activated haploidentical NK cells. Six patients achieved objective responses, out of which five patients proceeded to allogeneic hematopoietic stem cell transplantation (HSCT) and three patients remained in long-term remission. High-risk clones were found to be reduced or eliminated. This study supports the use of haploidentical NK cell infusions as a bridge to HSCT in refractory patients [19•]. Hence, it is evident that the anti-leukemic effect of NK cells may be further made more potent through activation of effectors or transfusion of activated effectors.

High doses of IL-2 are typically needed to achieve in vivo NK cell expansion with IL-2 as the only stimulatory cytokine, which may induce significant toxicity in patients. Low-dose IL-2 is not feasible given that it significantly expands T-regulatory cell numbers which end up limiting NK cell functionality. Hence, alternative treatment strategies utilizing IL-15 have been tested [20]. Recently, one group demonstrated in eight children with poor prognosis AML, B cell precursor ALL, and T-ALL that the infusion of ex vivo haploidentical CD3/CD19-depleted stem cell grafts (containing high numbers of NK cells) stimulated with IL-15 was safe and feasible [20].

There has been a focus of interest on NK-92, a transformed cell line fully comprising activated NK cells. This cell line is relatively easy to transfect and can be easily expanded under good-manufacturing-practice (GMP) conditions. Utilizing this cell line and redirecting these cells to specifically recognize tumor-associated surface antigens is gaining interest. For example, NK-92 cells have been used in ALL by transvecting the anti-CD19 CAR. These cells have been found to overcome the usual resistance of ALL cells to NK cell-mediated cytolytic activity, making NK-92 CAR an attractive potential target [21•]. Currently, there are only a handful of registered in-human clinical trials in CAR-NK cells [21•]. Most of these trials are being conducted in China using the CAR-engineered NK92 cells. The only trial using primary NK cells is being conducted in the USA at the MD Anderson Cancer Center (NCT03056339).

Overall, adoptive transfer of mature allogeneic NK cells in the nontransplant or transplant setting has been shown to be safe and feasible.

### NK Therapy in AML

Acute myeloid leukemia cells are more sensitive to NK-mediated cytotoxicity than solid tumor.

There have been several modifications attempted in order to boost the efficacy of NK cells. A first-in-man study investigated the role of memory-like NK cells, obtained after in vitro differentiation from human NK cells with IL-12, IL-15, and IL-18 [22, 23]. Additional manipulation of haploidentical NK cells such as priming with tumor lysate has also been studied in a phase I clinical trial in high-risk AML patients, with possibly some clinical efficacy observed [24]. An attempt to improve survival in intermediate-risk patients in first remission by adding haploidentical KIR-HLA mismatched NK cells as consolidation, however, did not increase overall survival or decrease relapse. Although this was tolerated, this result was also partly attributed to low cell dose and short life span in the recipient’s system [25]. They did suggest, however, that this does not preclude possible benefit of haploidentical NK cells if given in other phases of treatment.

### NK Therapy in ALL

Similar to T cells, NK cells can be modified to express CARs that recognize a variety of tumor antigens and these have been tested in vitro or in animal models with varying degrees of success [2, 4•, 10]. There are several advantages of CAR-NK over CAR-T cells. Firstly, NK cells disappear rapidly after mediating their anti-cancer effects, hence usually do not have long-term toxicity and should not require a ‘suicide system’ for elimination. Furthermore, even if cancer cells could evade immune surveillance by CAR cells via down-modulation of CAR-antigen target expression, NK cells would still be effective compared to T cells which would not be able to work at all. Also, while the cytokine-release syndrome that results from the activation of T cells can be severe and life-threatening, the cytokine profile from NK cells is usually clinically much milder. Lastly, there is no risk of GVHD from NK cells [27, 28].

Building on the grounds and knowledge gained with CAR T cells, a multitude of preclinical studies have tested the efficacy of CAR NK cells against a variety of target antigens for hematological malignancies apart from CD19, including CD3, CD20, and CD138 [24, 27, 29].

However, in terms of clinical development and in-human trials, it is fairly limited so far, with mainly two trials (NCT00995137 and NCT01974479) investigating NK cells expressing anti-CD19 CAR for the treatment of B lineage acute lymphoblastic leukemia [30, 31].

New investigations have been made into NK cells derived from human-induced pluripotent stem cells (iPSCs) that express this CAR. These NK-CAR-iPSC-NK cells have a typical NK cell phenotype which demonstrate an in vivo activity similar to that of T-CAR-expressing T cells, but with less toxicity, making them a potentially very suitable immunotherapy tool in leukemia. These NK-CAR-iPSC-NK cells are potentially able to provide standardized, targeted “off-the-shelf” lymphocytes for anti-cancer immunotherapy [32•].

## Conclusion

NK cell therapy can be further improved by optimal donor selection based on phenotypic and genotypic properties, by adoptive transfer of NK cells with ex vivo or in vivo cytokine stimulation, by the use of antibodies to induce antibody-dependent cellular cytotoxicity or to block iKIRs, or by transduction of chimeric antigen receptors.

## Natural Killer Cells in Solid Tumors

Infiltrating NK cells are known to be present in environment of solid tumors and metastases which to a certain extent control their growth and confer a favorable prognosis [4•]. In contrast to hematologic malignancies, NK cells have not seen the same success in solid tumors. This is due to the microenvironment where infiltrating cells and the tumor itself are immunosuppressive to NK cells. The tumor can secrete cytokines such as TGF beta that can suppress NK cell function but sustain innate lymphoid cells that have no cytotoxic capabilities.

### Neuroblastoma, Sarcomas, and Brain Tumors

#### Neuroblastoma

Use of anti-GD2 antibody in a chemotherapy backbone is now considered standard of care for high-risk neuroblastoma. NK cells play a pivotal role in the ways in which monoclonal antibodies to GD2 mediate its anti-tumor effects; however, in heavily pre-treated patients, NK cell killing may be adversely affected. Ch14.18 has been shown to activate NK cells and overcome KIR-mediated inhibition by specific HLA antigens [33]. However, efficacy is still subject to patient-specific KIR receptor, HLA ligand, and Fc receptor genotypes, which has been shown to have impact on the responses of the various anti-GD2 antibodies (hu14.18-IL-2 immunocytokine) 3F8, dinutuximab & ch14.18/CHO [34–37].

A more recent approach has been the use of haploidentical NK cells selected based on non-inhibitory KIR-HLA interactions. In newly diagnosed patients with high-risk neuroblastoma who were induced upfront with humanized anti-GD2 antibody and chemotherapy, consolidation with haploidentical NK cells has been tested in a phase 2 trial [38•, 39]. In this study, patients were consolidated with autologous hematopoietic cell transplantation after Busulfan/Melphalan conditioning; they were given further anti-GD2 antibody, granulocyte-macrophage colony-stimulating factor (GM-CSF), and IL-2, with or without haploidentical NK cells which were infused 2–5 days later. Long-term results including event-free survival are still under study after this phase 2 clinical trial demonstrated acceptable tolerability and feasibility.

In heavily pre-treated patients with recurrent or refractory disease, several approaches involving the timing of haploidentical NK cell infusion and chemotherapy with anti-GD2 antibody have been explored in clinical trials. In one approach, humanized anti-GD2 antibody, GM-CSF, IL2, and chemotherapy were administered in combination with haploidentical NK-cell infusions in alternating cycles and showed good responses in a pilot study [38•], the long-term follow-up results of which are eagerly awaited [38•]. In another approach for patients treated with 3F8, lymphodepleting cyclophosphamide-based chemotherapy was given prior to haploidentical NK cell infusion, which was then followed on with 3F8 therapy [40]. Using this approach, the overall response rate was 29% and patients who had received more than 10 × 10^6 NK cells/kg showed improved progression-free survival (HR: 0.36, 95%CI: 0.15–0.87, *p* = 0.022). Haploidentical NK cells have also been used through an approach with haploidentical transplant and treatment with ch14.18/CHO post transplant [41•], in a new donor-derived immune system. In this study, the 3-year OS was 58% with an EFS of 45%, with 7% transplant-related mortality in this otherwise very difficult to treat population, which demonstrates the feasibility and potential efficacy of this approach.

Expanded and activated haploidentical NK cells are also currently being studied in combination with various anti-GD2 antibodies, including ch14.18/CHO (ClinicalTrials.gov Identifier: NCT03242603) as well as hu14.18-IL2 (ClinicalTrials.gov Identifier: NCT03209869).

Autologous expanded NK cells are also being tested with Ch14.18 in combination with lenalidomide, a molecular analog of Thalidomide used in multiple myeloma which has been shown to stimulate NK cells and could potentially enhance ADCC through this immunomodulatory effect (ClinicalTrials.gov Identifier: NCT02573896).

From another perspective, TGFβ has been found to play an important role as a potent immunosuppressant in the tumor microenvironment of neuroblastomas, with high expression of TGFβ and TGFβR genes in patient samples. An emerging approach has been geared towards overcoming TGFβ-driven immunosuppression through TGFβ signaling blockade with Galunisertib [42] as well as the development of “armored” NK cells with a genetically modified TGFβ receptor [43], rendering them immune to the effects of TGFβ. This approach would potentially allow NK cells to infiltrate the tumor microenvironment better and to also improve cytolytic activity especially when combined with anti-GD2 antibody, and further clinical studies in patients are warranted.

#### Sarcomas

In pre-clinical in vitro studies, Ewing sarcoma and rhabdomyosarcoma were found to be exceptionally sensitive to NK cells expanded by coculture with K562-mb15-41BBL cells [44], which led to further phase I/II clinical trials using haploidentical NK cells that are currently ongoing (ClinicalTrials.gov Identifier: NCT02409576).

NK cell cytotoxicity against advanced rhabomyosarcoma has also been previously studied in the context of allogeneic stem cell transplants [43]. A retrospective analysis of 30 patients with advanced rhabdomyosarcoma from the European Group for Blood and Marrow Transplantation (EBMT) registries showed 3-year OS of 20% with a median survival time of 12 months; however, this analysis was limited due to the retrospective study design, heterogeneity of the study population, and that cell doses of NK cells were not available for analysis.

The graft versus tumor effect mediated by NK cells in haploidentical stem cell transplants have also been studied in small series and reports [44, 45]. In a small pilot study of six patients with NB, Ewing Sarcoma, Desmoplastic tumor, nasopharyngeal carcinoma, and embryonal rhabdomyosarcoma, half remained alive and in complete remission in a limited median follow-up of 14 months, suggesting a possible beneficial effect albeit limited by the size of study and heterogeneity of disease conditions [46].

With regard to NK cells and their role in ADCC, Enoblituzumab is a fully humanized Fc-optimized antibody against B7-H3 that is expressed on a wide range of solid tumors including sarcomas and is currently being studied in patients with CD276^+^ tumors, including sarcomas [47–49]. Other strategies that have been reported in pre-clinical studies involve the use of Smac mimetics, which are small molecules that have been shown to be able to sensitive tumor cells while activating NK cells and increasing their cytotoxicity [50].

#### Brain Tumors

While significant research is underway in adult glioblastoma multiforme, there has been limited progress in the use of NK cells as a platform for immunotherapy in childhood brain tumors even though studies of the tumor microenvironment in children suggest that they may be more amenable to NK cell cytotoxicity [51]. There are emerging studies of ^19^F-labeled NK cells in the treatment and localization of NK cell activity for medulloblastoma, the commonest childhood brain tumor, which can potentially lead to further progress in this field [52]. There are also other ongoing studies involving local injection of ex vivo expanded autologous NK cells via an ommaya reservoir for children with posterior fossa brain tumors (ClinicalTrials.gov Identifier: NCT02271711), as well as non-myeloablative haploidentical stem cell transplantation in phase II trials (ClinicalTrials.gov Identifier: NCT02100891); however, preliminary results are still unavailable.

#### Challenges in Solid Tumors

One of the main barriers to effective immunotherapy strategies in solid tumors is that of the immunosuppressive tumor microenvironment due to the presence of myeloid-derived suppressor cells (MDSCs) that affect cell homing, infiltration, and cytotoxicity of targeted cell-based therapies such as CAR-T cells. A new approach to overcome this involves the genetic modification of NK cells with a chimeric receptor to target and eliminate MDSCs [53•]. These modified NK cells are also programmed to secrete pro-inflammatory cytokines in otherwise immunosuppressive tumor microenvironments, to improve susceptibility to cytotoxic CAR-T cells. This approach can potentially be applied to a multitude of diseases where targeted cell therapies are already available but are hindered by the suppressive tumor microenvironment.

A model of lung metastasis suggests the immune suppression of NK cells’ ability to contain tumor cells with loss of cytotoxicity and expression of co-inhibitory molecules PD-1, LAG-3, and TIGIT. Use of IL2 and checkpoint inhibitors restored NK cell function [54]. Platelets in contact with cancer cells can inhibit NK cell function by producing RANKL for which Denosumab, a RANKL-neutralizing antibody, can restore function [55]. Platelets can coat cancer cells thus hiding them from NK cells.

## Enhancing NK Cell Function

To increase efficiency and overcome barriers to its function, methods to enhance activity have been explored. Allogeneic NK cells can be activated and expanded to increase in number and potency prior to infusion to the recipient. Interleukin administration (IL12, IL15, IL18) can increase the number and activity of NK cells. T_reg_ lymphocyte depletion can enhance NK cell function as they compete for IL2.

Chimeric antigen receptor using T cells (CAR-T) have received much attention and received FDA approval for B cell leukemias. In CAR therapy, immune effector cells are genetically modified to express receptors that recognize specified antigens on tumor cells and cause cell death. The cells contain an extracellular antigen receptor (scFv derived from a monoclonal antibody) and an intracellular signaling domain. Disadvantages in the use of T cells for CAR therapy include cytokine storm and persistence in the system which can cause chronic off target toxicities such as B cell aplasia seen in CD19 CAR. The short life span of NK cells that can limit its efficacy is an advantage for CAR therapy in that chronic toxicities can be avoided. Tumor cells can downregulate MHC receptors escaping T cells but will make them visible to NK cells. CAR NK cells are more powerful than regular NK cells. They can be transduced to produce IL-15 to increase life span in the system. Incorporation of a suicide gene that can be pharmacologically activated prevents persistence in the system once the desired effect is achieved [26]. The need for autologous harvest of T cells for CAR therapy is hindered by host immunosuppression from previous treatment and possible contamination with tumor cells. Allogeneic T cells, however, carry the risk for GVHD which is not seen with NK cells.

Antibodies against inhibitory receptors can restore activity. IPH2101 (I-7F9) targets KIR2DL-1, KIR2DL-2, and KIR2DL3 and Monalizumab (IPH2201) blocks NKG2A. They are being explored for use in combination with other treatments [56].

Targeting inhibitors of effector cells is one mechanism of increasing tumor cell kill activity. Immune checkpoint inhibition such as in PDL1 blockade used in combination with standard treatment augments tumor control by T cells. Monoclonal antibodies created to target receptors bearing ITIM domains such as NKG2A can enhance NK cell function in combination with PDL1 blockade [57].

## Conclusion

New insights into the biology of NK cells have led to further understanding of their role in immune surveillance. This has been important in refining the use of NK cells for adoptive cell therapy and in transplant. Enhancement of NK cell functions such as with the use of interleukins and engineering for CAR therapy provides a potent treatment option in select cancers in children. However, much of this knowledge still needs to be translated into the clinical setting. NK cell therapy and biology play a role in childhood leukemias and solid tumors. Although results in solid tumors seem inferior, the increasing knowledge on biology and behavior in cancer opens new options for refining treatment.
